# Crystal Violet Adsorption on Eco-Friendly Lignocellulosic Material Obtained from Motherwort (*Leonurus cardiaca* L.) Biomass

**DOI:** 10.3390/polym14183825

**Published:** 2022-09-13

**Authors:** Giannin Mosoarca, Cosmin Vancea, Simona Popa, Mircea Dan, Sorina Boran

**Affiliations:** Faculty of Industrial Chemistry and Environmental Engineering, Politehnica University Timisoara, Bd. V. Parvan, No. 6, 300223 Timisoara, Romania

**Keywords:** crystal violet, eco-friendly adsorbent, *Leonurus cardiaca* L., optimal adsorption conditions, adsorption isotherm, kinetic model

## Abstract

The performance of a new eco-friendly adsorbent, obtained from motherwort (*Leonurus cardiaca* L.) biomass after minimum processing, in crystal violet dye removal from aqueous solutions was studied. Firstly, the adsorbent material was characterized using several technics, such as FTIR, pH_PZC_ determination, SEM and color analysis. The next step was to determine the influence of initial dye concentration, contact time, temperature, pH, adsorbent dose and ionic strength on adsorbent adsorption capacity. Equilibrium, kinetic, thermodynamic, optimization and desorption studies were performed in a batch system for studying all aspects related to the adsorption process. The sips isotherm best fit the experimental data with a predicted maximum adsorption capacity of 125.6 (mg g^−1^). The kinetic data indicate that equilibrium is reached at 50 min and that general order is the best kinetic model to describe the dye retention. The process is endothermic, spontaneous, favorable and supposed to be a physical adsorption. In addition to establishing the optimal adsorption conditions, Taguchi methods and ANOVA analysis showed that the pH is the most influencing parameter of the adsorption process, having a contribution of 61.64%. All the presented data show that the motherwort biomass powder is very suitable to be used as at low-cost, easy available and effective adsorbent for the crystal violet dye removal from aqueous solutions.

## 1. Introduction

Dyes represent a class of compounds that have been used since ancient times. In the beginning, dyes were obtained from natural products of plant and animal origin. The obtaining process was difficult and achieved with low efficiency. The shade range was limited and the color fastness on the applied substrate was relatively low [1,2].

With the development of chemistry, humans gradually moved to obtaining synthetic dyes. Currently, synthetic dyes are the most widely used due to their very wide color palette and increased resistance to various external agents’ actions [2,3,4].

The large number of dyes used in the textile, paint, plastic, leather, cosmetic, pharmaceutical and food industries ultimately leads to large volumes of wastewater in which they are present. The removal of dyes from wastewater is essential because these compounds are toxic, carcinogenic and mutagenic [1,5,6,7,8,9].

One of the most commonly used dyes is crystal violet. It is widely used in the textile, paint and printing ink industries. It also has applications in human and veterinary medicine, where it is used as a disinfecting agent, coloring agent or animal drug. It is a toxic, mutagenic and carcinogenic substance that can cause skin and eye irritation, respiratory and kidney failure, increased heart rate, cyanosis and cancer. If it reaches natural effluents, even in low concentrations, it can inhibit the photosynthesis of aquatic plants and can affect aquatic organisms. It is nonbiodegradable and has a high chemical stability. Therefore, this dye must be removed from the wastewater before discharge [4,5,6,7,10].

Many methods such as: chemical and electrochemical oxidation, photochemical and photocatalytic processes, membrane separation, coagulation-flocculation, electrocoagulation, adsorption, precipitation, ion exchange and biological degradation are used for dye removal from water [1,5,6,9,10,11,12,13,14,15,16]. In many cases, adsorption is the preferred treatment process because it has several essential advantages: high efficiency, simplicity, selectivity, does not require special equipment with high energy consumption, and generally, the costs are low [3,6,8,9,11,15,16,17,18,19,20,21].

The costs related to adsorbent material represent approximately 70% of the adsorption process’ total costs [8,18]. This led to the search for economical adsorbent materials, which have low cost and can be used as such or after minimal treatment: industrial and agricultural wastes, mineral and vegetal natural materials. Many vegetal wastes and biomasses have proven to be effective adsorbents for the retention of dyes. These materials are very cheap and are easily available in large quantities and some are used to remove crystal violet from wastewaters: pineapple (*Ananas comosus*) crown leaves, pecan pericarp (*Carya illinoensis*), *Terminalia arjuna* sawdust, pineapple leaf powder, pecan nutshell, para chestnut husk, araucaria bark, palm cactus, mountain soursop seeds, *Ocotea puberula* bark, almond shells, *Citrullus lanatus* rind, *Moringa oleifera* pod husk, water hyacinth and formosa papaya seeds [3,8,9,11,15,16,19,20,22,23,24,25]. They contain cellulose, hemicellulose, lignin, polyphenols and proteins that have different functional groups (hydroxyl, carboxyl and amino), which makes them able to bind with dyes [12,13,26,27].

Motherwort (*Leonurus cardiaca* L.) is a herbaceous, perennial plant that grows to 1.5 m in large areas, such as Asia, Europe and North America. The stem is quadruple-edged and hairy, and the leaves are palmately lobed, similar to goose paws. It grows wild in meadows, woodlands, along roadsides, next to fences and on uncultivated lands in the plains and hills. It has been known since ancient times by Greeks, Romans and Chinese, for its beneficial effects on the heart, which explains the Latin name of the plant. Motherwort has antibacterial, antioxidant, anti-inflammatory and analgesic properties and is used for heart and blood circulation problems, and also, for neurological, gynecological and thyroid disorders [28,29,30].

The major advantage of this material over similar others reported in the literature is that this plant has been known since antiquity and grows freely on a large scale on all continents of the Northern Hemisphere, unlike other plants that are grown on a smaller scale or only on certain continents.

The main objective of this research was to demonstrate the potential of motherwort (*Leonurus cardiaca* L.) biomass as a new eco-friendly and low-cost adsorbent for the removal of crystal violet dye from aqueous solutions. Initially, the adsorbent materials were characterized using FTIR, SEM and color analysis. The influence of the main parameters that intervene in dye adsorption was analyzed. Kinetics, equilibrium, thermodynamics, optimization and desorption studies were also carried out.

## 2. Materials and Methods

The aerial parts of mature motherwort (*Leonurus cardiaca* L.) plants were acquired from StefMar (Ramnicu Valcea, Romania), which deals with the processing of traditional medicinal plants. After being washed with distilled water (one washing cycle in solid:liquid ratio = 1:10) and dried at room temperature (18–22 °C) for 5 days, the plants were placed in the oven at 105 °C for 24 h. The dried plant material was grounded electrically and passed through a 2 mm sieve.

The adsorbent material was characterized by SEM (FEI Inspect S model microscope, Eindhoven, The Netherlands) and FTIR (Shimadzu Prestige-21 spectrophotometer, Shimadzu, Kyoto, Japan) spectroscopies and color analysis in the CIEL*a*b* system (Cary-Varian 300 Bio UV-VIS colorimeter, Varian Inc., Mulgrave, Australia), while the point of zero charge was established using the solid addition method [31]. The SEM was performed in a low vacuum using a large field detector (LFD) at a cathode voltage of 25 kV and a working distance of approximately 10.8 mm. The FTIR sample was prepared by mixing the adsorbent with KBr and then pressing it into the appropriate pellet-forming shape. The spectrum was recorded in the range of 4000–450 cm^−1^.

The adsorption experiments were carried out in a batch system, at a constant mixing intensity (100 rpm), in three independent replicates for each experiment. Dilute solutions of HCl and NaOH (0.1 N) were used for pH adjustment, while the influence of ionic strength was investigated using NaCl as a background electrolyte. The crystal violet concentration was determined at a wavelength of 590 nm using a Specord 200 PLUS UV-VIS spectrophotometer (Analytik Jena, Jena, Germany).

The kinetic and equilibrium studies were performed at pH = 6, with an adsorbent dose of 2 (g L^−1^), at a temperature of 293 K and ionic strength of 0 (mol L^−1^) varying the contact time and initial dye concentration, respectively. The influence of the pH solution on adsorption capacity was studied at an initial dye concentration of 50 (mg L^−1^), adsorbent dose of 2 (g L^−1^), contact time of 50 min, temperature of 293 K and ionic strength of 0 (mol L^−1^). The influence of the adsorbent dose on adsorption capacity was determined at pH = 6, initial dye concentration of 50 (mg L^−1^), contact time of 50 min, temperature of 293 K and ionic strength of 0 (mol L^−1^), while the influence of ionic strength at pH = 6, initial dye concentration of 50 (mg L^−1^), contact time of 50 min, adsorbent dose of 2 (g L^−1^) and temperature of 293 K.

The dye amounts adsorbed at equilibrium (*q_e_*) were calculated with Equation (1):

(1) $$q_{e}=\frac{(C_{0}-C_{e})\cdot V}{m}$$

where *C*_0_ represents the initial dye concentration; *C_e_* represents the dye equilibrium concentration; *V* represents the solution volume and *m* represents the mass of the bioadsorbent.

Four isotherms (Langmuir, Freundlich, Temkin and Sips) and four kinetic models (pseudo-first order, pseudo-second order, Elovich and general order) were tested to study the equilibrium and kinetics of adsorption [7,13,25,32,33]. The equations of these models used to process the experimental data are presented in Tables S1 and S2, Supplementary Material. The appropriate adsorption isotherm and kinetic model were determined using the values of determination coefficient (R^2^), sum of square error (SSE), chi-square (χ^2^) and average relative error (ARE), whose equations are detailed in Table S3, Supplementary Material [33]. The experimental results obtained at 282, 293 and 307 K were the basis of the thermodynamic parameters’ calculation. Their specific equations are listed in Table S4, Supplementary Material [33,34].

The Taguchi method was developed to improve the efficiency of the dye removal process. An L27 orthogonal array experimental design (6 factors at 4 levels) was used to establish the optimal adsorption conditions. The analysis of variance (ANOVA-general linear model) was used to calculate each factor’s percentage contribution to the dye removal efficiency and to evaluate the results obtained by the Taguchi method. The necessary mathematical calculations were achieved with Minitab 19 software (version 19.1.1, Minitab LLC, State College, PA, USA).

The desorption experiments were carried out using three regenerating agents (distilled water, 0.1 N HCl and 0.1 N NaOH) in a batch system at constant stirring for 2 h. The calculation equation of the desorption efficiency is described in Table S5, Supplementary Material.

## 3. Results and Discussion

### 3.1. Adsorbent Characterization

#### 3.1.1. FTIR Analysis

**Figure 1:**
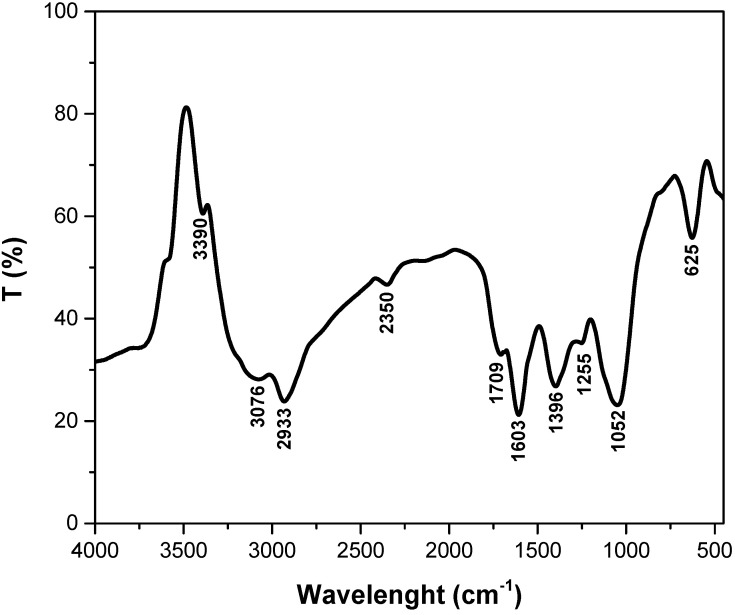
The FTIR spectra of adsorbents obtained from motherwort (*Leonurus cardiaca* L.) biomass.

The FTIR spectrum of adsorbents is presented in Figure 1. The peaks recorded in this spectrum, corresponding to different functional groups, show that the main components of the adsorbent material obtained from motherwort biomass are cellulose, hemicellulose and lignin. The identified peaks can be assigned as follows: 3390 cm^−1^—OH-stretching vibration related to cellulose and hemicellulose [35,36]; 3076 cm^−1^—aromatic C-H group [37]; 2933 cm^−1^—asymmetric stretching vibrations of C-H (methyl and methylene) related to cellulose, hemicellulose and lignin [36]; 2350 cm^−1^—aromatic ring C=C [38]; 1709 cm^−1^—conjugated C=O bonds [39]; 1603 cm^−1^—stretching vibration of C=C plus the asymmetric-stretching vibration of COOH in the aromatic ring related to cellulose, hemicellulose and lignin [40]; 1396 cm^−1^–both C-O stretch and O-H deformation in carboxylic acids [41]; 1255 cm^−1^—C–O stretching and CH or OH bending related to hemicellulose structures [42]; 1052 cm^−1^—stretching vibrations of C-O and C-C in cellulose, hemicellulose and lignin [31,36]; 625 cm^−1^—bending modes of aromatic compounds [43].

#### 3.1.2. Point of Zero Charge (pH_PZC_) Determination

**Figure 2:**
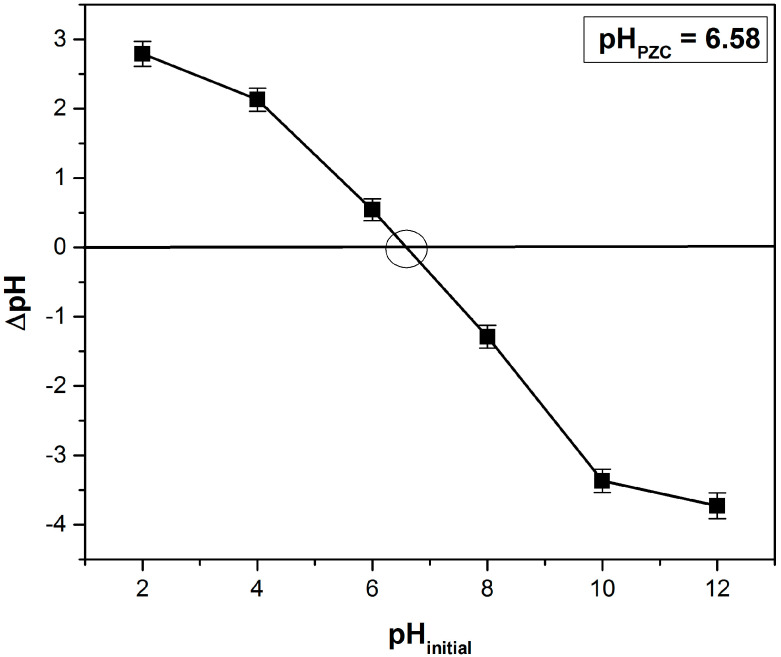
Determination of point of zero charge (pH_PZC_) based on the solid addition method.

The point of zero charge, pHpzc, is an important parameter that characterizes the surface of an adsorbent material. It indicates the pH value at which the net charge of the adsorbent surface is zero. For the motherwort biomass powder, the determined value was pH_PZC_ = 6.58 (Figure 2). At pH values under pH_PZC_, the adsorbent surface is positively charged and at pH values over pH_PZC_, the adsorbent is negatively charged. In the case of crystal violet dye adsorption, the solution pH > pH_PZC_ will favor the process. Scientific literature indicates that close values for pH_PZC_ were obtained for other similar adsorbents, such as 6.85 for formosa papaya seed powder [25], 7.1 for *Eragrostis plana* nees [34], 7.2 for Araticum seed powder [7] and palm kernel fiber [12] and 5.8 for cedar cone [4].

#### 3.1.3. SEM Analysis

**Figure 3:**
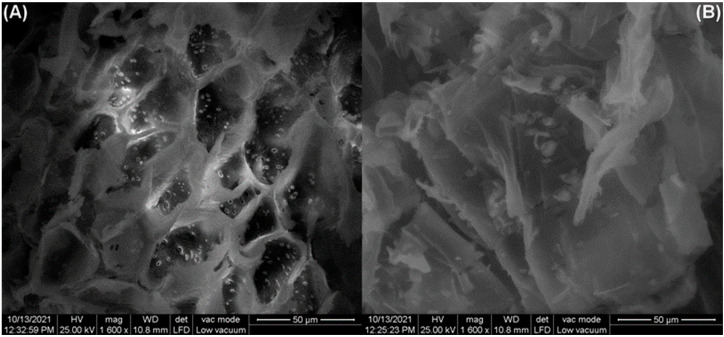
SEM images of adsorbent obtained from motherwort (*Leonurus cardiaca* L.) biomass before adsorption (**A**) and after adsorption (**B**).

Figure 3 shows the SEM images of the adsorbent surface before and after dye adsorption. Initially, the adsorbent surface is heterogeneous, presenting a microstructure having many irregularities and pores, susceptible to acting as adsorption sites (Figure 3A). After adsorption, the surface is drastically changed, less cavernous and more regular, suggesting that the adsorbed dye molecules filled the available pores and covered the surface irregularities (Figure 3B). 

#### 3.1.4. Color Analysis

**Figure 4:**
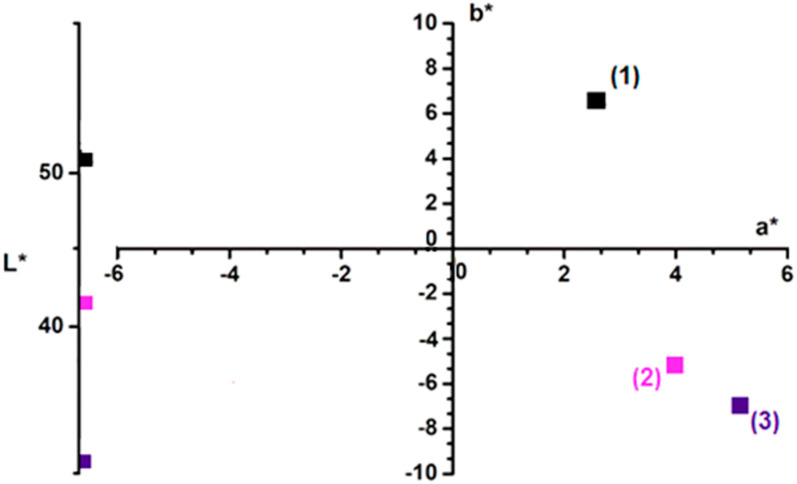
CIEL*a*b* color parameters of: (1) adsorbent before adsorption, (2) adsorbent after adsorption and (3) crystal violet dye.

To emphasize the adsorption process, the color analysis of the adsorbent material (before and after adsorption) was monitored with the CIEL*a*b* color parameters (Figure 4). The adsorption process permits the dye color in the wastewater to pass to the adsorbent material. Initially, the color of the adsorbent is described by Point (1). After adsorption, the material luminosity (L*) decreases and the values of a* and b* parameters change. Point (2), which describes the color of the adsorbent material after absorption, can be found in the color quarter of crystal violet (Point (3) describes the color of this dye). Thus, color analysis confirms the dye adsorption on the motherwort biomass powder.

### 3.2. Equilibrium Isotherms

**Figure 5:**
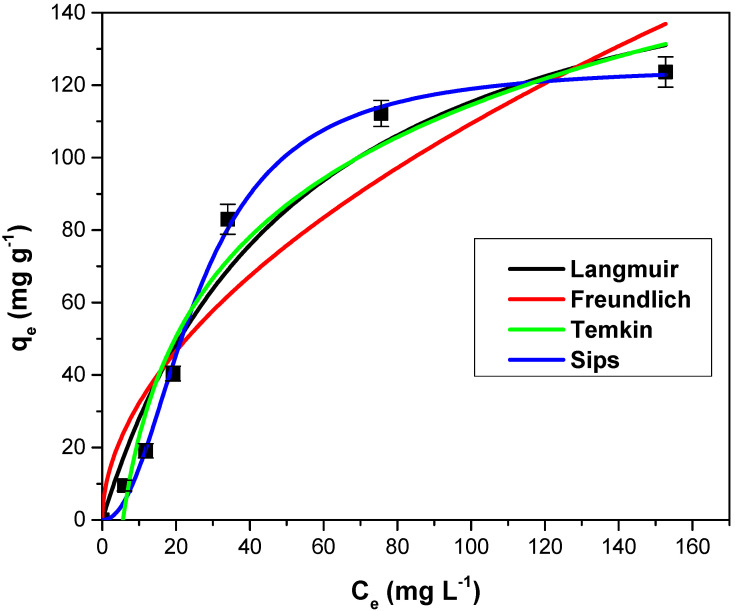
The experimental data and the fitted isotherm curves for crystal violet adsorption on adsorbents obtained from motherwort biomass.

The experimental isotherm data obtained for crystal violet dye adsorption on adsorbent material obtained from motherwort biomass are presented in Figure 5. Langmuir, Freundlich, Temkin and Sips isotherm models were fitted in order to obtain more information about the adsorption process at equilibrium. Analyzing the fitted isotherm curves, the obtained values of these isotherm constants and the corresponding statistical parameters (Table 1), it was concluded that the Sips isotherm is the suitable model to characterize the dye adsorption (higher value for R^2^, lower values for SSE, χ^2^ and ARE).

Table 2 shows the values of the maximum absorption capacities of some absorbents obtained from vegetable biomass, in the case of the retention of the crystal violet dye. The motherwort biomass powder has a better adsorption capacity, higher than other similar adsorbents.

### 3.3. Kinetic Models

**Figure 6:**
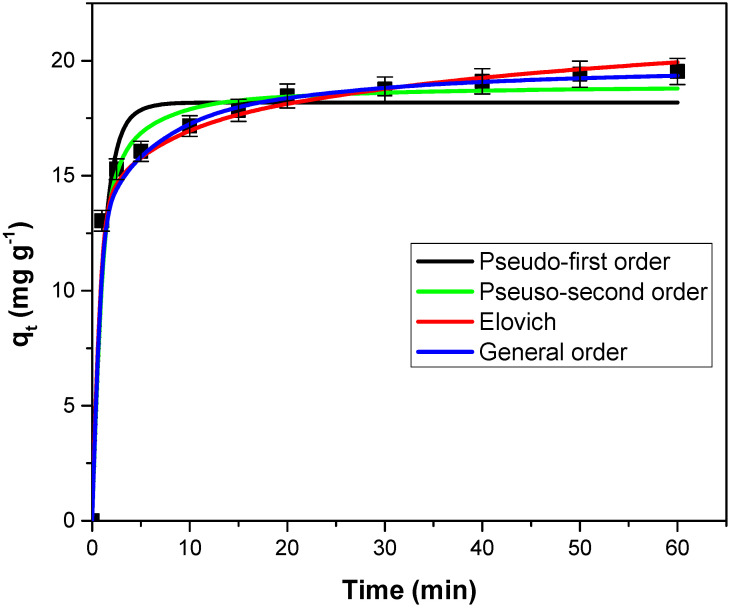
The experimental data and the fitted kinetic models curves for crystal violet adsorption on adsorbents obtained from motherwort biomass.

Figure 6 describes the influence of contact time on the adsorption capacity of the motherwort biomass powder. The adsorption capacity progressively increases until the equilibrium is reached at 50 min. In the first few minutes, adsorption capacity increases rapidly and with the passage of time, the increase is slower until equilibrium. This behavior can be explained by the fact that, in the first stage of the process, a high number of adsorption sites are available for dye retention. These sites are gradually occupied until equilibrium is reached, when it is considered that the surface of the adsorbent is practically completely covered by crystal violet molecules [7,9,12,23,27,36,59].

The scientific literature indicates that for the crystal violet dye adsorption on similar adsorbents, the following equilibrium times were obtained: approximately 30 min for *Arundo donax* L. [50]; approximately 50 min for *Lysiloma Latisiliquum* seed [48]; approximately 60 min for *Moringa oleifera* pod husk [23], papaya seeds powder [25], *Calotropis procera* leaf [44] and *Syzygium cumini* leaves [55]; approximately 120 min for *Ocotea puberula* bark powder [19], water hyacinth root powder [24], *Punica granatum* shell [27], corn stalk [46], pinus bark powder [52] and jackfruit leaf powder [56]; and approximately 180 min for *Eragrostis plana* nees [34].

The adsorption kinetics were investigated using four kinetic models. Their fitted curves are illustrated in Figure 6, and the constants and the corresponding statistical parameters are detailed in Table 3. The values of R^2^, SSE, χ^2^ and ARE indicate that the general order kinetic model best describes the adsorption process. 

### 3.4. Thermodynamic Study

The thermodynamic parameters of the adsorption process (Table 4) were calculated based on the experimental data obtained at 282, 293 and 307 K and those obtained from Figure S1, Supplementary Material. The standard Gibbs free energy change (ΔG^0^) has negative values and varies with increasing temperature, indicating a spontaneous and favorable process. The standard enthalpy change (ΔH^0^) and standard entropy change (ΔS^0^) have positive values. Therefore, the process is endothermic and at the solid-liquid interface appears to increase randomness (the degrees of freedom of the adsorbed species) [9,20,21,24,34].

The adsorption process is mainly physical adsorption, with van der Waals interaction having an important role [20,60]. At the same time, the value of ΔG^0^ is between −80 and −20 (kJ mol^−1^), indicating that in addition to physisorption, chemical adsorption may be involved in the process [42,61]. The value of ΔG^0^ closer to −20 (kJ mol^−1^) indicates that there is a small chemical effect that may enhance the process.

### 3.5. Influence of the pH Solution on Adsorption Capacity

**Figure 7:**
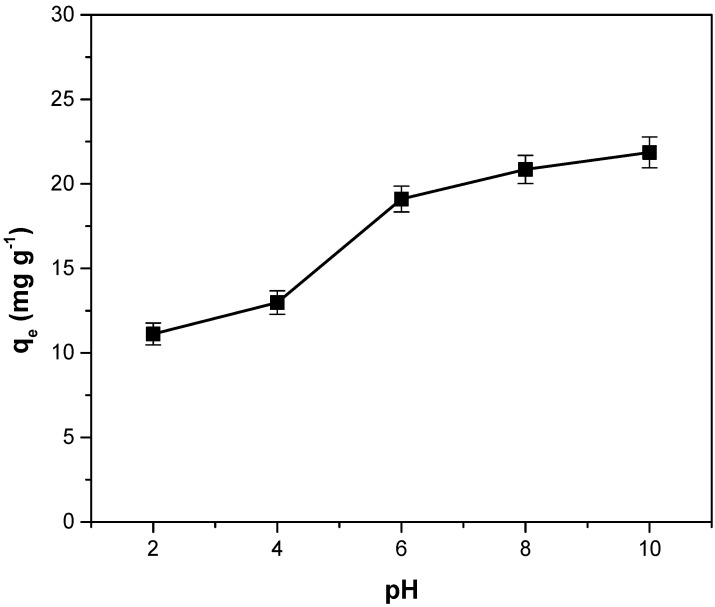
The influence of the pH solution on adsorption capacity for crystal violet adsorption on adsorbents obtained from motherwort biomass.

The variation in the adsorption capacity with the pH solution is illustrated in Figure 7. Increasing the pH value leads to an increase in the adsorption capacity. Similar results were reported for crystal violet dye adsorption on Araticum seed powder [7], pineapple leaf powder [11], *Ocotea puberula* bark powder [19] and *Moringa oleifera* pod husk [23]. The better performance is obtained at a pH higher than pH_PZC_ (6.58) and is generated by the electrostatic attraction that appears between the dye cations and the negatively charged adsorbent surface [7,11,19,54].

### 3.6. Influence of Ionic Strength on Adsorption Capacity

**Figure 8:**
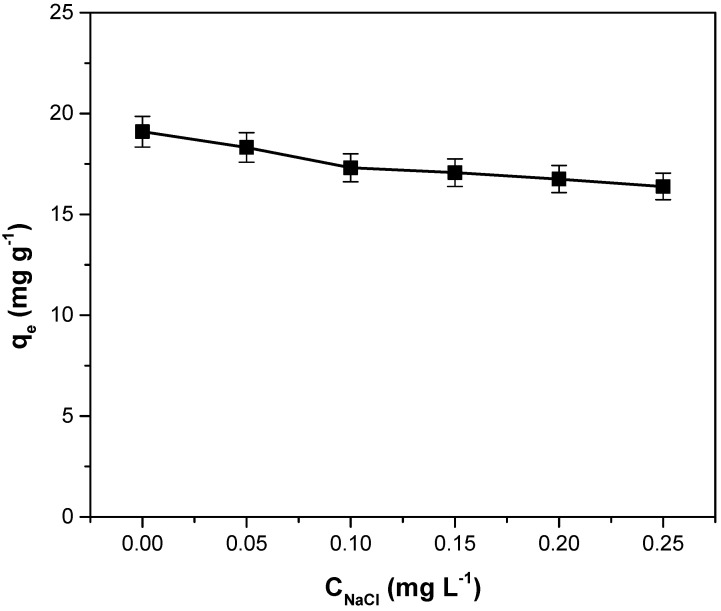
The influence of the ionic strength on adsorption capacity for crystal violet adsorption on adsorbents obtained from motherwort biomass.

The presence of different ions in the solution (characteristics of residual effluents) can influence the dye adsorption process. According to Figure 8, which shows the influence of the ionic strength on adsorption capacity, increasing the ionic strength leads to a decrease in the adsorption capacity. The adsorption process is affected by Na^+^ ions that compete with dye cations to occupy the adsorption sites on the surface of the motherwort biomass adsorbent. The same effect of ionic strength on adsorption capacity was mentioned in other similar studies [50,61,62].

### 3.7. Influence of Adsorbent Dose on Adsorption Capacity

**Figure 9:**
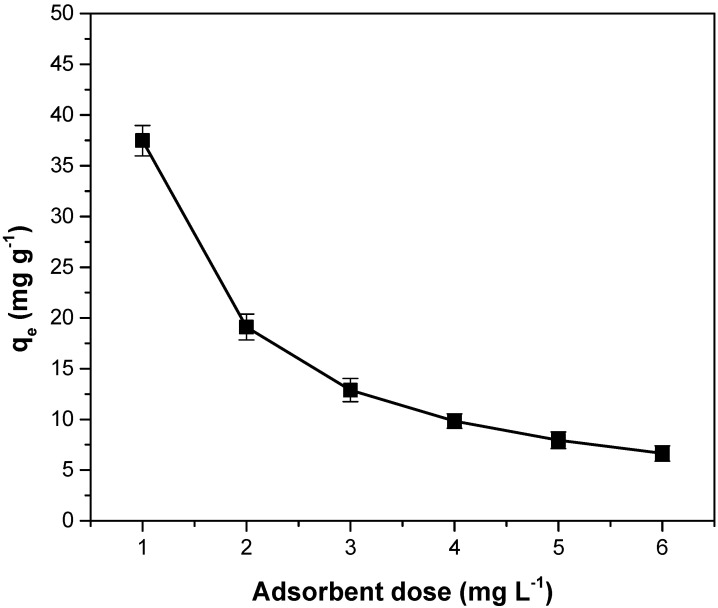
The influence of the adsorbent dose on adsorption capacity for crystal violet adsorption on adsorbent obtained from motherwort biomass.

As shown in Figure 9, the adsorption capacity decreases as the dose of added adsorbent material increases. Increasing the dose leads to an increase in the absorption surface and implicitly increases the number of the adsorption sites. However, a large part of them remains unsaturated. Additionally, agglomeration and aggregation phenomena of adsorbent particles may occur as the dose increases. All these aspects determine the decrease in the adsorption capacity [9,13,17,46]. Similar results regarding the influence of adsorbent dose on crystal violet adsorption were obtained for pineapple crown leaves [3], Araticum seed powder [7], *Terminalia arjuna* sawdust [9], pará chestnut husk [13], *Ocotea puberula* bark powder [19], *Eragrostis plana* nees [34], Anatolian black pine [47] and pinus bark powder [52].

### 3.8. Optimization Using the Taguchi Method

The optimal adsorption conditions were established using the Taguchi method, based on an L27 orthogonal array experimental design. The six factors and their levels that were the basis of this array are presented in Table 5.

The Taguchi approach focused on obtaining the signal-to-noise ratio (S/N) and analyzing it to evaluate the quality of the experiment and the validity of the result. The S/N ratio indicates the variability and level of precision for each response obtained in each experiment. The signal represents the response obtained by changing each operational factor, and the noise represents any factor that affects the precision. These are correlated with the value of the operational variable. The main advantages of this method are minimizing the number of experiments and visualizing the optimal conditions. To improve the dye removal efficiency, the “larger is the better” option for S/N ratio was taken into consideration [5,63,64,65,66]. The experimental results obtained for dye removal efficiency and the corresponding S/N ratios for each run are detailed in Table 6.

The S/N ratio for each factor at each level and the factors’ significance ranks (Table 7) show that pH was the factor with the highest influence on crystal violet removal efficiency, while the lowest impact was given by temperature. The optimal adsorption conditions determined with the Taguchi approach were: an initial dye concentration of 25 (mg L^−1^), contact time of 60 (min), temperature of 307 K, pH 10, an adsorbent dose of 6 (g L^−1^) and an ionic strength of 0.0 (mol L^−1^).

**Figure 10:**
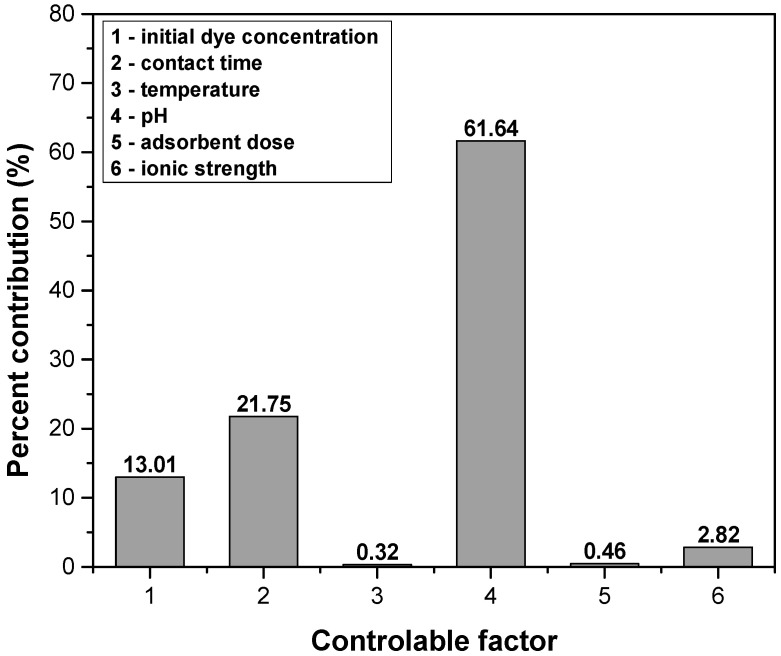
The contribution percentage of controllable factor influence on crystal violet removal efficiency based on ANOVA analysis.

The ANOVA analysis results are depicted in Figure 10 and indicate the contribution percentage of each controllable factor influencing the dye removal efficiency. Their values show the same order of controllable factors influenced by the Taguchi method.

**Figure 11:**
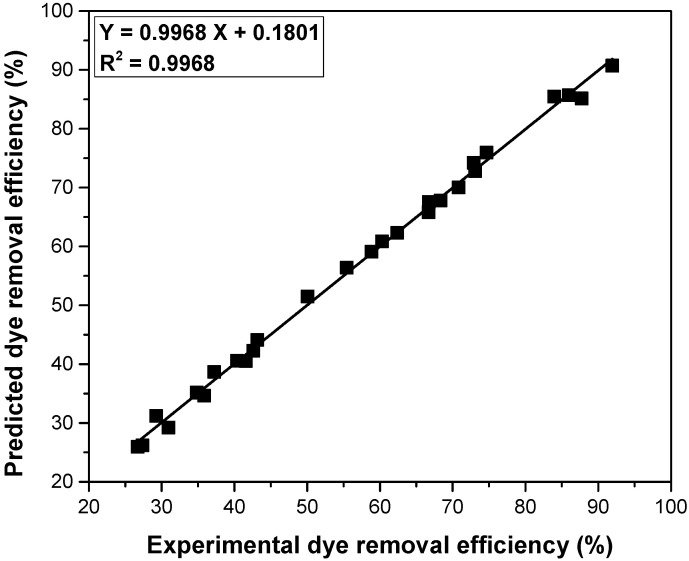
The correlation between the experimental values of dye removal efficiency with the predicted ones with optimization.

The accuracy of the Taguchi experimental design was verified by correlating the experimental values of the dye removal efficiency with the predicted ones with optimization (Figure 11). The straight line obtained with linear regression, the distribution of points and the value of R^2^ show high accuracy and indicate that the Taguchi method is very suitable for the optimization of the adsorption process.

### 3.9. Desorption Study

The results of the desorption study are detailed in Table S5, Supplementary Material. They show that significant values for the desorption efficiency were not obtained for the tested desorption agents. Therefore, regeneration of the adsorbent material is not recommended. Even if at first sight this is a disadvantage, it must be taken into account that the adsorbing material is very cheap, requires minimal processing and is found every year in nature in large quantities. The adsorbent material resulting from adsorption can also have practical utility. It is a vegetable material and has good combustion properties. Thus, in the context of the circular economy concept, it can be burned in specialized incinerators or can be used as a foaming agent in the thermal process of obtaining porous glass-ceramic materials and cellular glasses.

## 4. Conclusions

The adsorbent materials obtained from the motherwort biomass have a porous structure with different functional groups able to bind crystal violet dyes. The SEM and color analyses performed before and after adsorption, indicate the dye retention on the adsorbent surface. The increase in initial dye concentration, contact time, temperature and pH leads to improve adsorption capacity. The Sips isotherm and the general order kinetic model best fit the experimental data and the maximum adsorption capacity of 125.6 (mg g^−1^) is better compared to other similar adsorbents used for crystal violet dye adsorption. Thermodynamic studies indicate an endothermic, spontaneous, favorable and physical adsorption process. The optimal adsorption conditions determined using the Taguchi method were: an initial dye concentration of 25 (mg L^−1^), contact time of 60 (min), temperature of 307 K, pH 10, adsorbent dose of 6 (g L^−1^) and ionic strength of 0.0 (mol L^−1^). It was established that pH is the parameter that most influences the adsorption process with a contribution of 61.64%. All the obtained results show that the motherwort biomass powder has great potential to be used as a cheap, easily available, eco-friendly and effective adsorbent for crystal violet dye removal from aqueous solutions.

## Figures and Tables

**Table 1:** The tested isotherms models’ constants and the corresponding statistical parameters.

Isotherm Model	Parameters	Value
Langmuir	K_L_ (L mg^−1^)	0.018 ± 0.001
	q_max_ (mg g^−1^)	176.6 ± 4.75
	R^2^	0.9646
	χ^2^	14.17
	SSE	607.2
	ARE (%)	22.63
Freundlich	K_f_ (mg g^−1^)	9.51 ± 1.43
	1/n	1.88 ± 0.25
	R^2^	0.9075
	χ^2^	29.32
	SSE	1465
	ARE (%)	30.29
Temkin	K_T_ (L mg^−1^)	0.17 ± 0.05
	b (kJ g^−1^)	61.46 ± 3.24
	R^2^	0.9681
	χ^2^	22.81
	SSE	489.2
	ARE (%)	51.09
Sips	Q_sat_ (mg g^−1^)	125.6 ± 5.34
	K_S_ (L mg^−1^)	0.003 ± 0.001
	n	2.14
	R^2^	0.9978
	χ^2^	3.64
	SSE	35.36
	ARE (%)	15.57

**Table 2:** The values of the maximum absorption capacities of some absorbents obtained from vegetable biomass.

Adsorbent	Maximum Adsorption Capacity (mg g^−1^)	Reference
*Calotropis procera* leaf	4.14	[44]
*Calligonum comosum* leaf	5.00	[45]
corn stalk	9.64	[46]
anatolian black pine	12.36	[47]
cedar cones	13.64	[4]
*Lysiloma Latisiliquum* seed	14.14	[48]
*Salvinia natans* powder	12.74	[49]
*Arundo donax* L.	19.60	[50]
*Platanus orientalis* leaf	25.88	[51]
pinus bark powder	32.78	[52]
peel of *Cucumis sativa* fruit	34.24	[53]
date palm leaves powder	37.73	[54]
*Syzygium cumini* leaves	38.75	[55]
jackfruit leaf powder	43.39	[56]
*Eragrostis plana* nees	60.10	[34]
coir pith	65.53	[57]
*Laminaria japonica*	66.64	[58]
pineapple leaf powder	78.22	[11]
papaya seeds powder	85.99	[25]
motherwort biomass	125.6	This study
breadfruit skin	145.80	[59]
*Moringa oleifera* pod husk	156.25	[23]
water hyacinth root powder	322.58	[24]

**Table 3:** The tested kinetic models’ constants and the corresponding statistical parameters.

Kinetic Model	Parameters	Values
Pseudo-first order	k_1_ (min^−1^)	1.091 ± 0.023
	q_e,calc_ (mg g^−1^)	18.18 ± 0.71
	R^2^	0.9566
	χ^2^	0.79
	SSE	13.78
	ARE (%)	14.61
Pseudo-second order	k_2_ (min^−1^)	0.097 ± 0.004
	q_e,calc_ (g mg^−1^ min^−1^)	18.95 ± 0.74
	R^2^	0.9880
	χ^2^	0.22
	SSE	3.78
	ARE (%)	3.15
Elovich	a (g mg^−1^)	0.617 ± 0.081
	b (mg g^−1^ min^−1^)	5982 ± 253
	R^2^	0.9973
	χ^2^	0.55
	SSE	0.98
	ARE (%)	10.35
General order	k_n_ (min^−1^ (g mg^−1^)n^–1^)	0.0008 ± 0.0001
	q_n_ (mg g^−1^)	19.91± 0.46
	n	3.34
	R^2^	0.9979
	χ^2^	0.04
	SSE	0.69
	ARE (%)	0.94

**Table 4:** The thermodynamic parameters for crystal violet adsorption on adsorbents obtained from motherwort biomass.

ΔG^0^ (kJ mol^−1^)			ΔH^0^ (kJ mol^−1^)	ΔS^0^ (J mol^−1^ K^−1^)
282 K	293 K	307 K		
−20.94	−21.79	−22.86	0.074	9.19

**Table 5:** Controllable factors and their levels, used in the Taguchi design.

Factor	Level 1	Level 2	Level 3
Initial dye concentration (mg L^−1^)	25	200	400
Time (min)	1	30	60
Temperature (K)	282	293	307
pH	2	6	10
Adsorbent dose (mg L^−1^)	1	3	6
Ionic strength (mol L^−1^)	0	0.10	0.25

**Table 6:** The experimental results obtained for crystal violet removal efficiency and the corresponding S/N ratios using the Taguchi L27 orthogonal array.

Initial Dye Concentration	Time	Temperature	pH	Adsorbent Dose	Ionic Strength	Removal Efficiency	S/N Ratio
25	1	282	2	1	0	29.26	29.32
25	1	282	2	3	0.1	27.36	28.74
25	1	282	2	6	0.25	26.72	28.53
25	30	293	6	1	0	73.08	37.27
25	30	293	6	3	0.1	68.34	36.69
25	30	293	6	6	0.25	66.74	36.48
25	60	307	10	1	0	91.94	39.27
25	60	307	10	3	0.1	85.97	38.68
25	60	307	10	6	0.25	83.96	38.48
200	1	293	10	1	0.1	60.28	35.60
200	1	293	10	3	0.25	58.85	35.39
200	1	293	10	6	0	70.83	37.00
200	30	307	2	1	0.1	42.61	32.59
200	30	307	2	3	0.25	41.6	32.38
200	30	307	2	6	0	50.07	33.99
200	60	282	6	1	0.1	74.66	37.46
200	60	282	6	3	0.25	72.89	37.25
200	60	282	6	6	0	87.72	38.86
400	1	307	6	1	0.25	35.85	31.08
400	1	307	6	3	0	43.13	32.69
400	1	307	6	6	0.1	40.35	32.11
400	30	282	10	1	0.25	55.44	34.87
400	30	282	10	3	0	66.7	36.48
400	30	282	10	6	0.1	62.39	35.90
400	60	293	2	1	0.25	30.95	29.81
400	60	293	2	3	0	37.24	31.42
400	60	293	2	6	0.1	34.83	30.83

**Table 7:** Response table for signal-to-noise S/N ratios (larger is better).

Level	Initial Dye Concentration	Time	Temperature	pH	Adsorbent Dose	Ionic Strength
1	34.83	32.28	34.16	30.85	34.15	35.15
2	35.62	35.19	34.50	35.55	34.42	34.29
3	32.80	35.79	34.59	36.86	34.69	33.81
Delta	2.81	3.51	0.43	6.01	0.55	1.33
Rank	3	2	6	1	5	4

## Data Availability

All the experimental data obtained are presented, in the form of table and/or figure, in the article and in the Supplementary Materials.

## Supplementary Materials

The following supporting information can be downloaded at: https://www.mdpi.com/article/10.3390/polym14183825/s1, Table S1: The nonlinear equations of the tested isotherms; Table S2: The nonlinear equations of the tested kinetic models; Table S3: The corresponding equations for the statistical parameters R2, SSE, χ2 and ARE; Table S4: The equations of specific thermodynamic parameters; Table S5: The desorption efficiencies of the tested desorption agents; Figure S1: Plot of ln K_L_ vs. 1/T for crystal violet adsorption on adsorbent obtained from motherwort biomass.
