# Supplementary material for: Crystal Violet Adsorption on Eco-Friendly Lignocellulosic Material Obtained from Motherwort (Leonurus cardiaca L.) Biomass
Source: Polymers (Basel). 2022 Sep 13;14(18):3825. doi: 10.3390/polym14183825 (PMC9504819; doi:10.3390/polym14183825)
Supplement: Supplementary file 1 [file polymers-14-03825-s001.zip › polymers-1909554-supplementary.pdf]

polymers

MDPI

Supplementary material

Crystal Violet Adsorption on Eco-Friendly Lignocellulosic Material Obtained from Motherwort (*Leonurus cardiaca* L.) Biomass

Giannin Mosoarca, Cosmin Vancea *, Simona Popa *, Mircea Dan * and Sorina Boran

[1] Faculty of Industrial Chemistry and Environmental Engineering, Politehnica University Timisoara, Bd. V. Parvan, No. 6, 300223 Timisoara, Romania

* Correspondence: cosmin.vancea@upt.ro (C.V.); simona.popa@upt.ro (S.P.); mircea.dan@upt.ro (M.D.) Tel.: +40-256-404-194 (C.V.); +40-256-404-212 (S.P.); +40-256404176 (M.D.)

Citation: Mosoarca, G.; Vancea, C.; Popa, S.; Dan, M.; Boran, S. Crystal Violet Adsorption on Eco-Friendly Lignocellulosic Material Obtained from Motherwort (*Leonurus cardiaca* L.) Biomass. *Polymers* **2022**, *14*, 3825. https://doi.org/10.3390/polym14183825

Academic Editor: Carmelo Corsaro

Received: 26 August 2022

Accepted: 8 September 2022

Published: 13 September 2022

Publisher's Note: MDPI stays neutral with regard to jurisdictional claims in published maps and institutional affiliations.

Copyright: © 2022 by the authors. Licensee MDPI, Basel, Switzerland. This article is an open access article distributed under the terms and conditions of the Creative Commons Attribution (CC BY) license (https://creativecommons.org/licenses/by/4.0/).

Table S1. The non-linear equations of the tested isotherms.

Isotherm models	Equation
Langmuir isotherm	$q_e = \frac{q_m \cdot K_L \cdot C_e}{1 + K_L \cdot C_e}$
Freundlich isotherm	$q_e = K_F \cdot C_e^{1/n_F}$
Temkin isotherm	$q_e = \frac{R \cdot T}{b} \cdot ln(K_T \cdot C_e)$
Sips isotherm	$q_e = \frac{Q_{sat} \cdot K_S \cdot C_e^n}{1 + K_S \cdot C_e^n}$

where: q_m and Q_{sat} represents the maximum absorption capacities; K_L, K_F, K_T and K_S represents the Langmuir, Freundlich, Temkin and Sips isotherms constants; $1/n_F$ represents an empirical constant indicating the intensity of adsorption; R represents the universal gas constant; T represents the absolute temperature; b represents Temkin constant which related to the adsorption heat; n represents Sips isotherm exponent.

Table S2. The non-linear equations of the tested kinetic models

Kinetic and isotherm models	Equation
Pseudo-first-order kinetic model	$q_t = q_e(1 - exp^{-k_1 \cdot t})$
Pseudo-second-order kinetic model	$q_t = \frac{k_2 \cdot t \cdot q_e^2}{1 + k_2 \cdot t \cdot q_e}$
Elovich kinetic model	$q_t = \frac{1}{a} ln(1 + a \cdot b \cdot t)$
General order kinetic model	$q_t = q_n - \frac{q_n}{[k_n \cdot (q_n)^{n-1} \cdot t \cdot (n-1) + 1]^{1/1-n}}$

where: q_t represents the dye amount adsorbed at time t; k_1, k_2 and k_n represents the rate constants of pseudo-first-order, pseudo-second-order and general order kinetic models; q_e and q_n represents the theoretical values for the adsorption capacity; a represents the desorption constant of Elovich model; b represents the initial velocity; n represents the general order exponent.

Table S3. The corresponding equations for the statistical parameters R^2, SSE, χ^2 and ARE.

Statistical parameter	Equation
Determination coefficient	$R^2 = 1 - \frac{\sum_{i=1}^{n} (y_{i,exp} - y_{i,mod})^2}{\sum_{i=1}^{n} (y_{i,exp} - \overline{y_{i,exp}})^2}$
Sum of square error	$SSE = \sum_{i=1}^{n} (y_{i,exp} - y_{i,mod})^2$
Chi-square	$\chi^2 = \sum_{i=1}^{n} \frac{(y_{i,exp} - y_{i,mod})^2}{y_{i,mod}}$
Average relative error	$ARE = \frac{100}{n} \sum_{i=1}^{n} \left| \frac{y_{i,exp} - y_{i,mod}}{y_{i,mod}} \right|$

where: $y_{i,exp}$ represents the experimental value; $y_{i,mod}$ represents the modeled value; $\overline{y_{i,exp}}$ represents the mean values, n represents the total amount of information.

Table S4: The equations of specific thermodynamic parameters.

Thermodynamic parameters	Equation
Standard Gibbs free energy change	$\Delta G^0 = -RT \ln K_L$
Standard enthalpy change<br>Standard entropy change	$\ln K_L = \frac{\Delta S^0}{R} - \frac{\Delta H^0}{RT}$

where: R represents the universal gas constant; K_L represents the Langmuir constant, T represents the absolute temperature.

Table S5. The desorption efficiencies of the tested desorption agents.

Desorption agent	Desorption efficiencies (%)
HCl	11.56 ± 1.21
distilled water	4.11 ± 0.76
NaOH	4.28 ± 0.87

Desorption efficiencies equation

$$D(\%) = \frac{m_d}{m_a} \cdot 100$$

where: m_d represents the dye amount liberated by the regenerating agent, m_a represents the dye amount adsorbed on the adsorbent material.

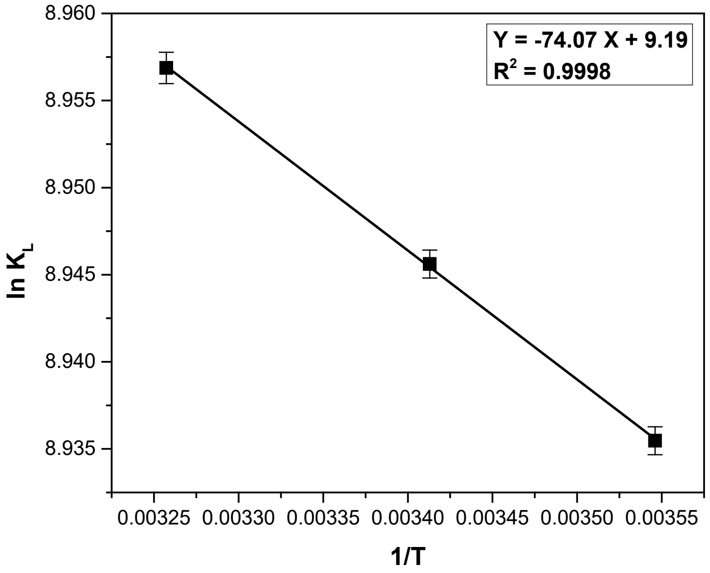

Figure S1. Plot of ln KL vs. 1/T for crystal violet adsorption on adsorbent obtained from motherwort biomass
